# Clinico-pathological features of diabetic and non-diabetic renal diseases in type 2 diabetic patients: a retrospective study from a 10-year experience in a single center

**DOI:** 10.1007/s11255-023-03478-4

**Published:** 2023-03-06

**Authors:** Yuemeng Sun, Yawei Ren, Ping Lan, Xiaoyang Yu, Jie Feng, Dapeng Hao, Liyi Xie

**Affiliations:** 1 Department of Nephrology, Xi’an People’s Hospital (Xi’an Forth Hospital), Xincheng District Jiefang Road 21, Xi’an, 710001 Shannxi China; 2 Department of Nephrology, The First Affiliated Hospital of Xi’an Jiaotong University, Yanta Western road 227, Xi’an, 710061 Shaanxi China

**Keywords:** Type 2 diabetes mellitus, Renal biopsy, Diabetic retinopathy, Membranous nephropathy, PLA2R antibody

## Abstract

**Aim:**

To compare clinical and pathological characteristics as well as prognosis between diabetic nephropathy (DN) and non-diabetic renal disease (NDRD) so as to explore potential diagnostic criteria of DN and provide some guidance for the treatment of type 2 diabetes mellitus (T2DM) patients with kidney involvement.

**Methods:**

T2DM patients with renal impairment who underwent kidney biopsy were included in this study, who were classified into 3 groups (DN, NDRD, DN with NDRD) based on their renal pathological diagnosis. Baseline clinical characteristics as well as follow-up data were collected and analyzed among 3 groups. Logistic regression was performed to determine the best predictors for DN diagnosis. Additional 34 MN patients without diabetes were enrolled by propensity score matching method to compare serum PLA2R antibody titer and kidney outcomes between diabetic MN patients and MN alone.

**Results:**

Among 365 patients with type 2 diabetes who underwent kidney biopsy, 179 (49.0%) patients were diagnosed with NDRD alone and 37 (10.1%) patients with NDRD combined DN. Risk factors for DN development in T2DM patients were longer time since diabetes diagnosis, higher level of serum creatinine, absence of hematuria and presence of diabetic retinopathy by multivariate analysis. Lower rate of proteinuria remission and higher risk of renal progression were observed in DN group compared with NDRD group. Membranous nephropathy was the most common NDRD in diabetic patients. There was no difference in serum PLA2R antibody positiveness or titer between MN patients with or without T2DM. There was lower remission rate but similar renal progression in diabetic MN when age, gender, baseline eGFR, albuminuria and IFTA score were adjusted.

**Conclusions:**

Non-diabetic renal disease is not uncommon in T2DM patients with renal impairment, which has better prognosis with proper treatment. Coexisting diabetic status does not exert negative impact on renal progression in MN patients, and immunosuppressive agents should be administered when necessary.

## Introduction

The incidence of diabetic nephropathy (DN) has dramatically increased recent years, becoming a significant cause for end-stage renal disease (ESRD) and posing great burden to healthcare system [1–4]. Patients with type 2 diabetic mellitus accompanied by kidney injury can present with following conditions: diabetic nephropathy(DN) alone, non-diabetic renal disease(NDRD) and DN combined with NDRD [5]. Typical appearances of DN include glomerular basement membrane thickening and K–W nodules formation. The prevalence of NDRD in T2DM patients with kidney injury as confirmed by kidney biopsy fluctuates between 12.3 and 69% [6, 7], depending on ethnic populations and different indications for renal biopsy. Membranous nephropathy is the most common NDRD in diabetic patients among Chinese population [8]. Renal prognosis of DN and DNRD remains controversial. In this study, we aimed to compare clinical presentation and prognosis between DN, NDRD, and DN combined with NDRD. Since MN is common among diabetic patients with NDRD, we aimed to investigate the differences in serum anti-PLA2R antibody and renal prognosis between patients with MN combined with diabetes and MN alone.

## Materials and methods

### Patients inclusion and data collection

Patients who diagnosed with T2DM with renal impairment who underwent kidney biopsy at first affiliated hospital of Xi’an JiaoTong University (Shannxi, China) from October 2010 to September 2019 were included in this retrospective study. Diagnosis of T2DM was made according to criteria by World Health Organization and the American Diabetes Association [9].The indications for renal biopsy were: sudden onset of heavy proteinuria, unexplained rapidly progressive renal failure, persistent glomerular hematuria and absence of diabetic retinopathy. Clinical parameters were collected at the time of renal biopsy including age, gender, time since diabetes diagnosis, presence of hypertension, and presence of diabetic retinopathy. Additional laboratory parameters, such as levels of serum creatinine, blood urea nitrogen, glycosylated hemoglobin, 24-h proteinuria, presence of glomerular hematuria were also collected. Patients were followed up until endpoints was met. Renal outcomes such as remission of proteinuria and kidney function progression were compared among there groups.

Since MN was the most common NDRD in diabetic patients, we aimed to find whether coexistence of diabetes would affect renal outcomes in MN patients. Therefore, additional 34 MN patients without T2DM were included to set as control group by propensity score matching method (age, gender, serum albumin, 24-h urinary protein, eGFR, IFTA score for kidney pathology evaluation were set as control variables). The differences of serum anti-PLA2R antibodies as well as renal outcomes were analyzed between patients of MN combined with diabetes and MN alone.

### Renal pathology

Patients were divided into 3 groups by pathological diagnosis as following: diabetic nephropathy(DN), non-diabetic kidney disease(NDRD) and NDRD combined with DN. Kidney biopsy sections were evaluated independently by 3 different pathologists. For light microscopic examination, all the specimens were processed with periodic acid Schiff, hematoxylin–eosin, methenamine silver and trichrome. Immunofluorescence staining was performed for detection of antibodies to IgG, IgA, IgM, C1q and C3. Electron microscopy was applied in all samples for ultra-structural investigations. Diagnosis of DN and pathological score for interstitial fibrosis and tubular atrophy (IFTA) was evaluated according to the criteria described by the Renal Pathology Society [10].

### Management protocol

Supportive care was given to all patients including glycemic control (either insulin or oral agents), angiotensin-converting enzyme inhibitors and/or angiotensin receptor blockers to control proteinuria and to maintain blood pressure ≤ 130/80 mmHg (proteinuria < 1 g/day) or ≤ 125/75 mmHg (proteinuria > 1 g/day), statins for hyperlipidemia, dietary salt restriction and diuretics if edema was present. Immunosuppressants were administered if necessary, and anticoagulant therapy were given to patients who were at high risk for thrombosis.

### Definitions

Hypertension was diagnosed by meeting any of the following criteria: (1) previous diagnosis of hypertension; (2) undergoing treatment for anti-hypertensive; (3) systolic blood pressure ≥ 140 mmHg, or diastolic blood pressure ≥ 90 mmHg during administration. Glomerular hematuria was defined as number of erythrocytes ≥ 3/HPF, and > 70% red blood cells are abnormal under phase contrast microscope. Serum levels of total IgG anti-PLA2R antibodies were measured by the ELISA test. Participants were considered as anti-PLA2R–positive when levels were > 20 RU/ml. Complete remission (CR) was defined as proteinuria less than 0.3 g/24 h with stable renal function. Partial remission (PR) was defined as more than 50% reduction in proteinuria of the baseline level and < 3.5 g/24 h with stable renal function [11]. No response (NR) was defined as proteinuria > 3.5 g/24 h or with decreased renal function. Renal insufficiency was defined as > 30% decline of eGFR or doubling of serum creatinine and eGFR < 60 ml/min/1.73m^2^.

### Statistics analysis

Continuous data were presented as mean ± SD(normally distributed data) or median with inter-quartile range (non-normally distributed data) and categorical data as frequency (%). Differences between groups were evaluated with Student *t* test or ANOVA for normally distributed data, with Mann–Whitney *U* test or Kruskal–Wallis test for non-normally distributed data and with chi-square (χ2)-test or Fisher exact for categorical data. Multiple logistic regression analysis was applied to determine independent predictors for DN diagnosis (age, gender, time since diabetes diagnosis, diabetic retinopathy, hematuria, baseline serum creatinine, and proteinuria quantification for 24 h were set as variables, “enter” method was applied for logistic regression). Cut-off value for time since diabetes diagnosis was defined as median value and cut-off value for serum creatinine was defined as upper limit of normal range. The cumulative incidence of ESRD was compared between groups by Kaplan–Meier analysis. A two-sides value of *P* < 0.05 was considered as statistically significant. All statistical analyses were performed by SPSS for Windows version 25.0 (IBM SPSS Statistic 25.0, September 2017. IBM Corporation. Chicago, USA).

## Results

### Pathological characteristics

#### DN

**Fig. 1:**
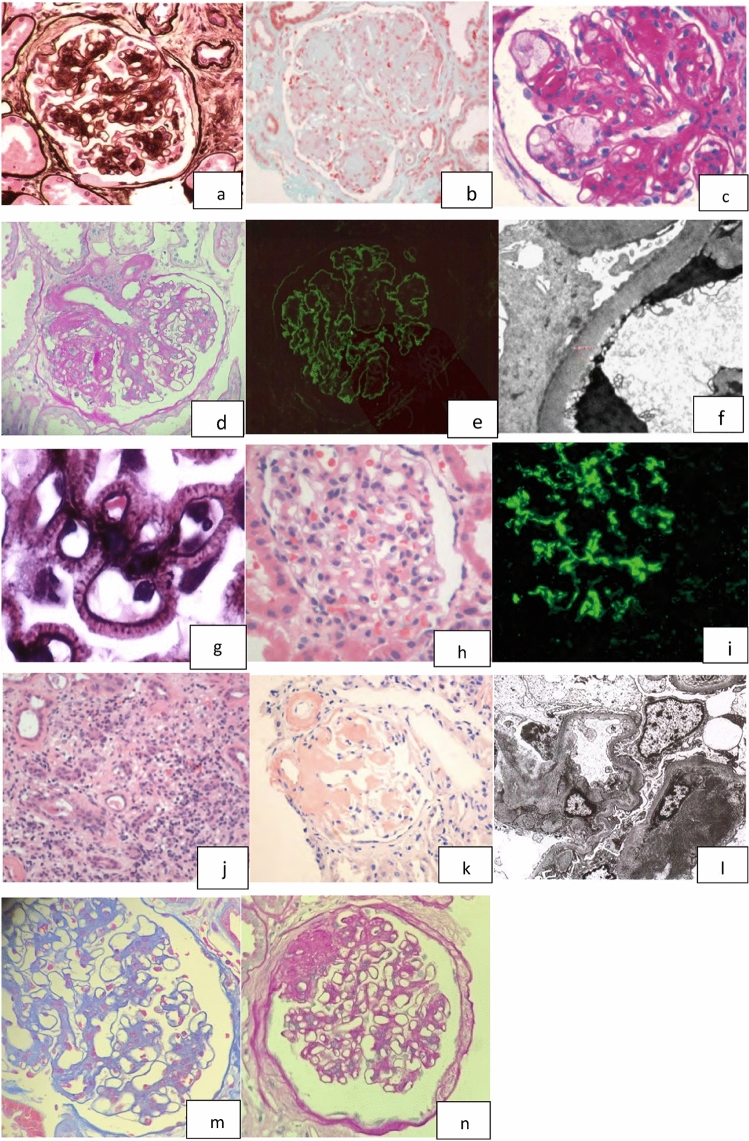
Pathological characteristics of DN and NDRD **a** stage II, diabetic nephropathy, Sliver staining, × 200 **b **stage III DN, Kimmelstiel–Wilson nodule, PAS staining, × 200 **c** stage III DN, glomerular capillary microangioma formation. PAS staining, × 400 **d** Hyalinization of the outgoing and incoming arteries. PAS, × 200 **e** IF, diffuse linear staining of glomerular basement membrane for IgG in diabetic nephropathy **f** EM, diffuse thickening of glomerular basement membrane without the presence of electron dense granules in DN **g** Membranous nephrology, GBM thickening, spike formation, deposition of pheoglobin subepithelium, Sliver staining, × 1000 **h** IgA nephropathy. PAS, × 200 **i** IF, deposition of IgA and C3 in mesangial area. **j** Acute interstitial nephritis, proximal tubular epithelial cells necrosis and inflammatory cell infiltration were presented. PAS, × 200 **k** AL amyloidosis nephropathy, Congo red staining. × 200 **l** EM, amyloid deposited along the capillary, presented as disordered fiber. **m** Mesangial proliferative glomerulonephritis imposed on DN: mesangial cell proliferation and mesangial matrix expansion can be seen. Masson's trichrome stain. **n** Primary FSGS imposed on DN, segmental GBM thickening, focal sclerosis and collapse capillary loops can be observed. Sliver staining × 200

One hundred and forty-nine patients (40.8%) were diagnosed with DN alone among 365 patients who underwent kidney biopsy. Typical lesions such as mesangial expansion, renal cystic droplet lesion (Fig. 1a) and Kimmelstiel–Wilson nodules (Fig. 1b) were observed in diabetic nephropathy. Vascular involvement in diabetic nephropathy presented as microangioma formation (Fig. 1c) and arterial hyalinosis (Fig. 1d). Immunofluorescence showed linear staining of glomerular IgG deposition. (Fig. 1e) Diffuse thickening of glomerular basement membrane without electron dense deposition was a typical pathological lesion in DN. (Fig. 1f).

#### NDRD

179(49.0%) cases were proved to have NDRD alone, of which membranous nephropathy (Fig. 1g) was the most common disease (56 patients; 31.3%), followed by mesangial proliferative glomerulonephritis (Fig. 1h)(47 patients; 26.3%) and IgA nephropathy (Fig. 1i) (38 patients; 21.2%). Disease spectrum of NDRD is listed in Table 1.

**Table 1:** NDRD identified in complicated group and non-DN group

Disease spectrum	NDRD (*n* = 179)%	DN + NDRD(*n* = 37)
Membranous nephropathy	56 (31.3)	9 (24.3%)
Mesangial proliferative glomerulonephritis	47 (26.3)	10 (27.0%)
IgA nephropathy	38 (21.2)	5 (13.5%)
Focal segmental glomerulosclerosis	19 (10.6)	5 (13.5%)
Acute interstitial nephritis	6 (3.35)	5 (13.5%)
ANCA-associated vasculitis	4 (2.23)	1 (2.7%)
Amyloidosis	3 (1.68)	1 (2.7%)
Henoch–Schonlein Purpura nephritis	2 (1.12)	0
Lupus nephritis	1 (0.56)	0
Membranous proliferative glomerulonephritis	1 (0.56)	1 (2.7%)
Others	2 (1.12)	0

Fifty six patients in NDRD group were diagnosed as primary membranous nephropathy, since secondary causes such as HBV infection, SLE or malignancy were absent and no immune complex deposition was found in glomerular mesangial area. Glomerular basement membrane thickening and spike formation were observed in cases of MN. Immunofluorescence showed granular staining for IgG and C3 along glomerular capillary, of which IgG subclass presented IgG4-dominant, with a few cases showed weaker IgG1 coexisted (figure not shown). Acute interstitial nephritis was one of less common causes of NDRD, where presented proximal tubular epithelial cells necrosis and inflammatory cell infiltration (Fig. 1j). In case of AL amyloidosis nephropathy, Congo red-positive amyloid fibril was found in mesangial area and artery vessels (Fig. 1k), which exhibited disordered fibers with diameters of 8–12 nm by electron microscopy (Fig. 1l).

#### NDRD + DN

In cases of membranous nephropathy combined with DN, diffuse thickening of glomerular basement membrane and spike formation were observed by silver staining. Severe mesangial expansion and nodular sclerosis could be seen in patients with MN combined with stage 3 diabetic nephropathy. A few cases presented chronic lesions such as interstitial fibrosis and tubular atrophy. By immunofluorescence, granular IgG deposition was observed and IgG4 was the dominant IgG subclass in most cases. Electron microscope revealed immune complex deposition in the subepithelium area without absorption vacuole, indicating early stages of MN in these cases. When mesangial proliferative glomerulonephritis combined with DN (Fig. 1m), mesangial cell proliferation was presented; while in diabetic nephropathy, cell proliferation was uncommon. In addition, electron dense deposition in mesangial area could be observed in mesangial proliferative glomerulonephritis combined with DN, but no immune complex deposition was noticed in DN.

In cases of IgA nephropathy combined with DN, mesangial expansion and immune complex deposition was present. Immunofluorescence findings showed strong IgA deposition in mesangial area. In cases of primary FSGS combined with DN, we observed segmental GBM thickening and mesangial cells proliferation which leading to collapse capillary loops in sclerosis area (Fig. 1n). The pathological difference between secondary FSGS caused by hyper-filtration and primary FSGS was that the former occurred at the vascular pole while the latter developed at random region within the glomerulus.

### Clinical characteristics and prognosis between DN and NDRD

Three hundred and sixty-five patients diagnosed with type 2 diabetes who had renal impairment and underwent renal biopsy were included, of which 149 patients (40.82%) had isolated DN, 179 patients (49.04%) had isolated NDRD and 37 patients (10.14%) had DN combined with NDRD.

Clinical characteristics among three groups (DN alone, NDRD alone and DN combined with NDRD) are summarized in Table 2. No differences were noticed in age, gender and HbA1c level. There was higher level of proteinuria and serum creatinine as well as higher incidence of diabetic retinopathy in DN group and DN combined with NDRD group. Time since diabetes diagnosis was longer in DN patients compared to NDRD group.

**Table 2:** Clinical characteristics of the patients in classification groups (isolated DN vs mixed lesions vs isolated NDRD)

	DN (*n* = 149)	NDRD (*n* = 179)	DN + NDRD (*n* = 37)	*P* value
Age (years)	54.74 ± 9.59	53.78 ± 11.0	53.08 ± 11.89	> 0.05^0^
Gender (male, *n*%)	102 (68.5%)	124 (69.3%)	28 (75.7%)	> 0.05^0^
Proteinuria (g/24 h)	3.77 ± 1.99	2.96 ± 2.04	3.89 ± 2.43	0.04^a^ (0.023^b^, 0.016^c^)
SCr(umol/L)	140.56 ± 102.00	110.98 ± 61.68	192.73 ± 98.18	0.004^a^ (0.002^b^, 0.034 ^c^)
BUN(mmol/L)	8.67 ± 3.44	7.35 ± 2.68	11.65 ± 13.13	0.002^a^ (0.011^c^, < 0.001^d^)
Time since DM diagnosis(years)	7.63 ± 5.25	3.64 ± 3.83	7.39 ± 5.46	0.009^a^ (0.01^b^)
HbA1c	7.05 ± 1.64	7.12 ± 1.35	7.45 ± 1.54	> 0.05^a^
Hypertension, *n*(%)	127 (85.23%)	85 (47.49%)	11 (39.73%)	0.001^a^ (< 0.001^b,d^ 0.048^c^)
Retinopathy, *n*(%)	121 (81.21%)	41 (22.91%)	20 (54.05%)	< 0.001^a^ (< 0.001^b,^^c^ 0.01^d^)
Hematuria, *n*(%)	43 (28.86%)	94 (52.51%)	20 (54.05%)	< 0.001^a^ (< 0.001^b^ 0.004^d^ > 0.05^c^)
Follow up data				
Rate of remission	6.7% (2/30)	46.6% (28/60)	21.4% (3/14)	< 0.001^a^ (< 0.001^b^ > 0.05^c,d^)
Rate of renal insufficiency	70.0% (21/30)	16.6% (10/60)	57.1% (8/14)	< 0.001^a^ (< 0.001^b^ 0.005^c^ > 0.05^d^)

*ANOVA* analysis of variance, *BUN* blood urea nitrogen, *Scr* serum creatinine, *DN* diabetic nephropathy, *NDRD* non‐diabetic renal disease

^a^ANOVA for continuous variables and Chi-square test for categorical variables

^b^Post hoc DN versus NDRD

^c^Post hoc NDRD versus DN + NDRD

^d^Post hoc DN versus DN + NDRD

**Fig. 2:**
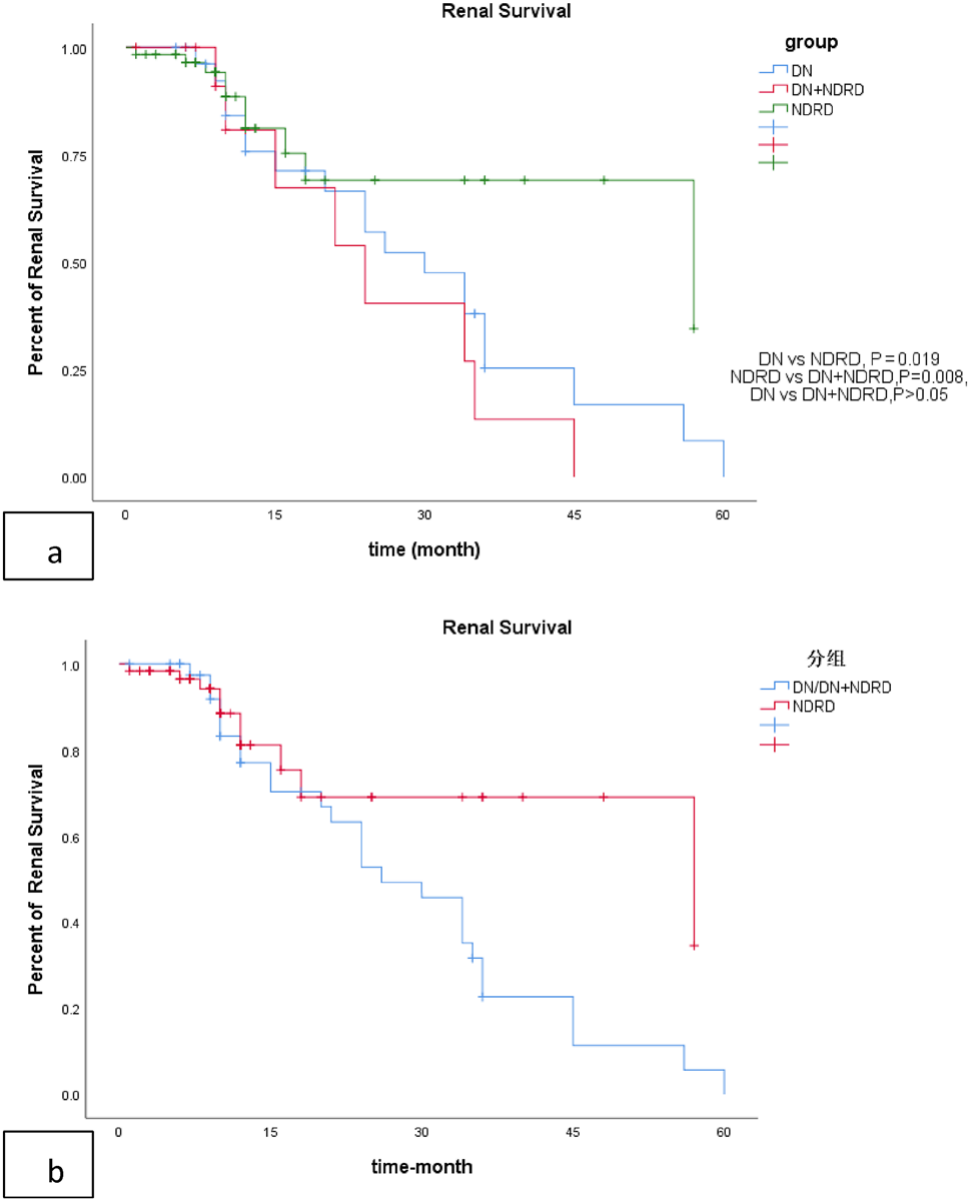
**a** Renal survival curve among group DN、NDRD、DN + NDRD. **b** Renal survival curve between group DN or DN combined with NDRD and group NDRD

After median 25 months of follow-up, 28 patients (46.6%) with NDRD achieved clinical remission (CR + PR), which was significantly higher than DN patients. Lower renal insufficiency rate (16.6% vs 70%, *P* < 0.001) was observed in NDRD group compared with DN group, while no difference was noticed between DN and NDRD + DN group. Detailed statistics are shown in Table 2. Kaplan–Meier analysis showed that patients with NDRD had lower probability for renal progression than DN patients or DN combined with NDRD patients (Fig. 2a, b).

Binary logistic regression analysis disclosed that serum creatinine > 97 umol/L (*P* < 0.001,OR:9.15, CI 95%:4.00–20.91), time since diabetes diagnosis > 60 months (*P* < 0.001, OR:2.16, CI 95%:1.11–4.19), absence of hematuria (*P* = 0.003,OR:2.92,CI 95%:1.43–5.99), presence of diabetic retinopathy (*p* < 0.001, OR:27.6,CI 95%:10.55–72.33) were independent predictors for diagnosis of diabetic nephropathy. Results of the multiple logistic regression analysis are shown in Table 3.

**Table 3:** Multi-variable logistic regression analysis for diabetic nephropathy as dependent variable

	B ± E	*P* value	Odds ratio	95% CI for Odds ratio
Male	–	> 0.05	1.44	0.56–3.68
Age > 65 y	–	> 0.05	1.15	0.39–3.33
SCr > 97 umol/L	2.213 ± 0.422	< 0.001	9.15	4.00–20.91
Proteinuria > 3.5 g/24 h	–	> 0.05	1.66	0.96–2.87
Presence of hypertension	–	> 0.05	0.42	0.1–1.06
Absence of Hematuria	1.072 ± 0.366	0.003	2.92	1.43–5.99
Time since diabetes diagnosis > 60 months	0.769 ± 0.338	0.023	2.16	1.11–4.19
Presence of retinopathy	2.162 ± 0.362	< 0.001	27.6	10.55–72.33

*SCr* serum creatinine, *CI* Confidence interval

### Comparison between MN and MN combined with T2DM

#### Anti-PLA2R antibody in MN combined with T2DM

Among 56 patients diagnosed with primary membranous nephropathy and diabetes, 34 patients had follow-up data available for prognostic analysis. Another 34 primary MN patients without diabetes were set as control group by PSM(propensity score matching)method(age, gender, serum albumin, urinary protein, eGFR, IFTA score for kidney pathology evaluation were set as control variables). Clinical characteristics were compared between MN alone and MN combined with T2DM. The rate of serum PLA2R-ab positivity in MN alone patients did not differ from those of MN with diabetes (64.7 vs 47.1%, *P* > 0.05). Besides, average antibody titer between 2 groups was not significantly different. (92.5 ± 146.6 RU/ml vs 112.2 ± 184.4 RU/ml, *P* > 0.05).

#### Treatment and prognosis in MN combined with T2DM

Thirty four patients diagnosed with MN combined diabetes were available for prognostic analysis, 18 of whom were treated with low-dose steroids or other immunosuppressants while other 16 cases were treated only with RAAS-inhibitors. On the contrary, 76.4% MN patients without diabetes were treated with immune-suppressive agents, a much higher rate compared with diabetic MN patients (*P* < 0.05). When age, gender, serum albumin, urinary protein, eGFR and IFTA score were adjusted at baseline, no difference in renal insufficiency rate was noticed between patients of MN alone and MN with diabetes (5.7 vs 14.7%, *P* > 0.05) Moreover, higher rate of proteinuria remission was achieved in MN without diabetes group at the end of follow-up (55.9% vs 29.4%, *P* = 0.027). Detailed statistics are shown in Table 4.

**Table 4:** Comparison of clinical characteristic and prognosis between MN and MN with diabetes

	MN without T2DM (*n* = 34)	MN with T2DM (*n* = 34)	*P* value
Sex(male/female)	26/8	26/8	> 0.05
Age (years)	54.1 ± 11.8	54.2 ± 9.2	> 0.05
Serum albumin (g/l)	22.2 ± 7.0	22.2 ± 4.9	> 0.05
Quantification for 24 h proteinuria (g/24 h)	4.9 ± 2.0	4.9 ± 2.3	> 0.05
Baseline eGFR (ml/min*1.73 m^2^)	89.6 ± 12.8	87.3 ± 27.2	> 0.05
IFTA score	0.25 ± 0.55	0.30 ± 0.73	> 0.05
Positivity for serum PLA2R antibody (%,*n*)	64.7% (22/34)	47.1% (16/34)	> 0.05
Baseline PLA2R antibody titter(RU/ml)	92.5 ± 146.6	112.2 ± 184.4	> 0.05
Rate of proteinuria remission (%,*n*)	55.9% (19/34)	29.4% (10/34)	0.027
Rate of renal insufficiency (%,*n*)	5.7% (2/34)	14.7% (5/34)	> 0.05
Rate of immunosuppression therapy (%,*n*)	76.4% (26/34)	52.9% (18/34)	0.042

For continuous variables *t *test or Mann–Whitney *U* test was applied, and for categorical chi-square or Fisher exact was applied

## Discussion

With high prevalence of DM all around the world, DM coexisting with chronic kidney disease is not uncommon. In this large retrospective research, we confirmed non-diabetic renal disease could develop alone or combined with DN in T2DM patients. Therefore, the present study was designed to explore the risk factors for DN diagnosis so as to provide some indications for kidney biopsy in diabetic patients with kidney injury when atypical symptoms presents. We confirmed several factors predicting the diagnosis of DN including diabetes vintage, absence of hematuria, presence of diabetic retinopathy and decreased eGFR at baseline. Development of diabetic nephropathy usually occurs 5–10 years after the onset of type 2 diabetes. Several researches confirmed that longer duration of diabetes was a risk factor for DN development [12–14]. However, the cut-off value for DM duration was not on agreement due to the difficulty of early diagnosis for T2DM with subtle symptom. We found that over 5 years since diabetes diagnosis was an independent risk factor for DN development.

It is believed that patients with diabetic nephropathy has lower eGFR [14, 15] compared to NDRD in diabetic patients. Vistisen et al. [16] found that diabetic patients experienced progressive eGFR decline even without albuminuria. Our results indicated higher level of creatinine was a risk factor for DN diagnosis. The fact that diabetic patients are often combined with various complications such as hypertension, hyperlipidemia and cardiovascular disease, may be a possible reason for their renal insufficiency.

We also found the absence of hematuria was independent factor for DN diagnosis. The mechanism of hematuria is more likely due to thinning or disruption of glomerular basement membrane such as in IgA nephritis or thin basement membrane disease. Therefore, presence of hematuria is unusual in diabetic nephropathy which often presents with thickening of GBM. Besides, Moreno [17] found close relationship between glomerular hematuria and glomerular inflammatory injury. Since DN is often considered as a non-inflammatory disease, presence of hematuria is helpful to differentiate NDRD from DN.

The association of diabetic nephropathy and diabetic retinopathy are commonly recognized [18, 19] since both diabetic nephropathy and diabetic retinopathy are complications for diabetic micro-vascular disease. Our results revealed a significantly higher rate of DR in DN group than NDRD group, and presence of DR was the strongest predictor for development of DN with odds ratio being 27.6. However, diabetic retinopathy was concordant with DN in only about 60 to 65% of diabetic patients [12], and additional risk factors such as absence of hematuria combined with proliferative DR would help to differentiate DN from NDRD.

There are controversial opinions on the prognosis between DN and NDRD. Lorenzo V et al. [20] found that the degree of renal decline was similar in patients with DN and NDRD after adjusting for baseline albuminuria, and concluded that higher albuminuria was associated with poorer renal outcome regardless of diabetic condition. While others [21, 22] agreed that kidney survival was better in NDRD patients despite the similar baseline eGFR in NDRD and DN groups, and the worst prognosis fell in patients with NDRD combined with DN. Similar to previous researches, our results revealed lower risk of renal progression in NDRD patients. The inferior prognosis in DN could be explained that hyperglycemic environment may lead to glomerular hyper-filtration as well as renal tubular ischemia and oxidative stress, thus leading to deceased renal function [22]. These results emphasized the great significance of kidney biopsy in T2DM patients with high suspicion for NDRD development since accurate diagnosis and appropriate treatment could greatly improve their prognosis.

Our research not only confirmed the presence of non-diabetic renal disease in more than half of diabetic kidney disease, but also presented the disease spectrum of NDRD in diabetic patients. There is no agreement on the incidence of NDRD in patients with T2DM, varying from 12 to 81% [23–26], probably due to distinctive races and regions as well as different criteria for kidney biopsy. It is reported that IgA nephropathy is the most common NDRD in Southeast Asia, while FSGS and AIN is the prevailing pathology in US and India, respectively [20, 27, 28]. We confirmed membranous nephropathy was the most common NDRD among diabetic patients in Chinese population. The high prevalence of membranous nephropathy in Chinese population could also be observed by other researches. HLA risk allele may be one of the pathogenesis of MN development [29, 30]. Other environmental factors such as air pollution, bacterial infections and occupational exposure might increase the risk of idiopathic MN as well [31, 32].

We wondered if the pattern of IgG subclass in kidney biopsy was different in MN patients with diabetes. As a result, dominant IgG4 deposition was observed in majority cases of MN combined with diabetes, consistent to the IgG subclass pattern in primary MN [33, 34]. We observed weak IgG1 with IgG4 deposition in a few cases. Huang et al. [36] showed IgG1-dominant deposition in early stages of MN, indicating an IgG subclass switch in the antibody response with IgG4 taking over later as the dominant immunoglobulin. Zhang et al. [37]revealed IgG1-dominant deposition in diabetic nephropathy and indicated that renal prognosis was influenced by IgG deposition. We speculated IgG1 deposition in present cases resulted from early stages in primary MN since the causes of secondary MN or renal lesion of diabetic nephropathy were absent. Besides, other autoantigens such as THSD7A, exostosin1/exostosin2, and NELL-1 may explain for heterogeneous IgG subclass distribution in PLA2R-unrelaed MN [37].

M-type phospholipase A2 receptor (PLA2R) is well believed to be a major auto-antigen in 70–80% primary MN patients, which also has close correlation to disease activity and prognosis [38, 39]. Research on the role of ani-PLA2R antibody in MN with diabetes is limited, and the diagnostic role of ani-PLA2R antibody for MN in T2DM patients remains equivocal. Research from a single center in China [40] indicated the optimal cut-off value of serum anti-PLA2R antibodies for diagnosis of IMN with diabetes was 2.71RU/ml. In present research, no difference was noticed regarding to positiveness of PLA2R antibody and PLA2R antibody titer in MN patients with or without T2DM. We speculated that PLA2R antibody still had potential diagnostic value in MN patients with diabetes, though the results should be interpreted with caution because of the limited cases in each group.

There is no agreement on whether coexisting diabetes affect the renal outcomes in MN patients. Therefore we carried out this study to analyze the impact of coexisting diabetes on remission rate and renal progression rate in iMN patients. There was opinions that hyperglycemic status caused renal hemodynamic disorder and oxidative stress [22, 41], which resulted in lower remission rate and renal progression. Domestic and international studies showed that baseline diabetes was associated with failure to achieve complete remission in MN patients [42, 43], independently of baseline renal function and therapeutic regimens. On the contrary, other results [42, 45]showed no inferiority in complete remission of diabetic MN patients compared to MN alone. Our findings were identical to the former researches, and we supposed less application of immunosuppressants in our cohort might be a possible explanation for lower remission rate. As for renal prognosis, we did not observe inferior renal prognosis in MN patients with diabetes when other risk factors for ESRD [45] such as eGFR, albuminuria and pathological IFTA score were controlled. These results provide some guidance on the treatment of MN patients with diabetes where immunosuppression agents, given as needed, may be helpful in kidney function preservation without obvious adverse effect on glycemic metabolism.

There are some limitations of this study. First, only type 2 diabetic adults with suspicion of NDRD were enrolled, so select bias could not be avoided. Second, cases with early stage of diabetic nephropathy could be missed due to absence of typical pathological changes. Third, due to limited samples of MN with diabetes and relatively short follow-up duration, we could not prove the relation between serum anti-PLA2R antibody level and clinical remission.

In conclusion, this relatively large retrospective research revealed some differences between diabetic nephropathy and non-diabetic nephropathy regarding to their clinical presentation and kidney prognosis. The fact that NDRD patients endure a better prognosis confirms the great significance of kidney biopsy in patients with atypical clinical presentation, which enables the individualized therapy in order to improve the prognosis of diabetic patients with NDRD. Furthermore, our results indicated that combined diabetes did not exert negative effect on renal prognosis in iMN patients who should be treated with immunosuppression therapy when necessary.

## Data Availability

All data generated or analyzed during this study are included in this published article and its supplementary information files.
